# A Review of Parental Vaccine Hesitancy for Human Papillomavirus in Japan

**DOI:** 10.3390/jcm12052004

**Published:** 2023-03-02

**Authors:** Madoka Lelliott, Ethan Sahker, Hemant Poudyal

**Affiliations:** 1 Department of Health Promotion and Human Behavior, Graduate School of Medicine/School of Public Health, Kyoto University, Kyoto 606-8501, Japan; 2 Population Health and Policy Research Unit, Graduate School of Medicine, Kyoto University, Kyoto 606-8501, Japan

**Keywords:** immunization, children, parents, cervical cancer, virus

## Abstract

Globally, Japan has the lowest rate of vaccine confidence. The persistent parental vaccine hesitancy has been attributed to safety and efficacy concerns and is primarily driven by the negative experience with human papillomavirus (HPV) vaccines. This literature review aimed to identify factors associated with HPV vaccine uptake and potential strategies to reduce vaccine hesitancy among Japanese parents. Articles published in English or Japanese between January 1998 and October 2022 that examined Japanese parental factors for HPV vaccine uptake were identified from PubMed, Web of Science, and Ichushi-Web. In total, 17 articles met the inclusion criteria. Four key themes which affected HPV vaccine hesitancy and acceptance were identified: perceptions of risk and benefits, trust and recommendation, information and knowledge, and sociodemographic characteristics. While governmental and healthcare provider recommendations are important factors, efforts to improve parental confidence in the HPV vaccine are required. Future interventions to counteract HPV vaccine hesitancy should actively disseminate information on vaccine safety and effectiveness, along with information on the severity and susceptibility of HPV infection.

## 1. Introduction

Vaccine hesitancy is defined as the “delay in acceptance or refusal of vaccination despite the availability of vaccination services” [1]. Despite a remarkable decline in vaccine-preventable diseases (VPD) due to aggressive national pediatric immunization programs in many countries [2,3,4], parental vaccine hesitancy remains a critical issue. In 2019, the World Health Organization identified vaccine hesitancy as one of the top 10 threats to global health and included it in its 5-year strategic plan [5].

Although the parental perception of vaccination varies by vaccine, a general pattern that determines parental vaccine decisions has been identified among high-income countries, which includes trust in healthcare providers and pharmaceutical companies, social networks, social norms, knowledge/sources of vaccine information, and risk perceptions regarding vaccines and VPD [6]. Despite being a high-income, high-education country, Japan has the lowest global vaccine confidence rate, primarily due to vaccine safety and effectiveness concerns among the general population [7]. As in other high-income countries, several outbreaks of VPD in Japan have recently been reported. For instance, major measles and rubella outbreaks were reported in 2018 in Japan and were attributed to vaccine hesitancy [8].

The human papillomavirus (HPV) is involved in the pathogenesis of cutaneous and anogenital warts and several types of cancer [9]. The cervix/uterus is the most common HPV-attributable cancer site representing over a third of all HPV-attributable cancer burdens globally, followed by the anus, vulva, vagina, oropharynx, and penis [10]. Other less common types of HPV-attributable cancer include esophageal, head and neck, lung, skin, and brain [9,10]. Consequently, older people and females have higher HPV-attributable cancer incidence rates than younger people and males [10]. While the age-standardized incidences of HPV-attributable cancer decreased by 16.7% between 1990 and 2012 globally, some African and Asian countries, notably Uganda (+46.4%), China (38.6%), and Japan (+18.9%), experienced an increase in the incidences of HPV-attributable cancer during this period [10]. Interestingly, Japan is one of the seven countries that reported a rise in HPV-attributable cancer incidence after an initial decline, the others being the United Kingdom, the Netherlands, Germany, Denmark, and Australia [10].

Regarding HPV vaccines, in Japan, Cervarix was approved in October 2007, and Gardasil 4 was approved in July 2010. These vaccines were included in the national immunization program for girls aged 12–16 in April 2013. In the program, the vaccination costs were fully covered by subsidies until the prescribed age. After starting the national immunization program, the HPV vaccination rate quickly rose to around 70% among girls aged 13–16 nationwide [11]. According to Japan’s Ministry of Health, Labour, and Welfare (MHLW), of the approximately 3.38 million people who took the HPV vaccine, 2584 (0.08%) were estimated to have experienced adverse events such as chronic pain and movement disorders by 2014 [12]. Of the 1739 people with available adverse event data, 1550 people (89.1%) recovered, and 186 people (10.7%) did not [12]; the non-recovered group represents about 0.005% of the total vaccinated population [12]. Nevertheless, the MHLW announced the withdrawal of proactive recommendations for HPV vaccination in June 2013 due to widespread media backlash [13]. As a result, the HPV vaccination rate dropped from around 70% at its peak to under 1% [14]. Simms et al. [15] estimated that 24,600–27,300 HPV cases and 5000–5700 deaths among girls born between 1994 and 2007 were caused by a lack of HPV vaccination and projected an additional 3400–3800 cases and 700–800 deaths by 2020. However, the actual incidence and mortality surpassed the estimates, with 10,978 diagnosed with cervical cancer in 2018 and 2921 deaths in 2019 [16]. Given this situation, the Japan Society of Obstetrics and Gynecology (JSOG) favored an early reinstating of the proactive vaccine recommendation. However, the MHLW did not resume a proactive recommendation of the HPV vaccine until November 2021. Additionally, although Gardasil 9 was approved in July 2020, parents must pay about JPY 30,000 (€216) per dose because there is no public expenditure for both boys and girls.

HPV vaccine withdrawal resulted in greater anti-vaccine sentiment in Japan, which also reverberated globally [17,18]. While extensive studies over the past decade within Japan and elsewhere have demonstrated the safety and effectiveness of HPV vaccination [17], these concerns continue to drive parental vaccine hesitancy [1,19,20,21,22]. Although the MHLW has resumed proactive recommendation, negative perception of the HPV vaccine persists among mothers, and the vaccine gap for HPV has emerged as a major public health problem in Japan [17]. This scoping review explores parental hesitancy for HPV vaccination in Japan and discusses potential areas of interest to address vaccine hesitancy.

## 2. Method

### 2.1. Search and Selection Procedure

This review was conducted according to the methodological framework for scoping reviews [23]. The search for articles was conducted using the PRISMA Extension for Scoping Reviews (PRISMA-ScR) Checklist [24]. A strategy was developed to identify literature published up to and including October 2022 on HPV vaccine hesitancy in Japan using PubMed, Web of Science, and Ichushi-Web (Igaku Chuo Zasshi; Japan Medical Abstracts Society) databases. The search was conducted in October 2022 using the search terms shown in Table 1. The inclusion criteria required the article to be an original report (reviews, editorials, commentaries, and letters to editors were excluded) published in English or Japanese, focused within a Japanese context, reporting on HPV vaccination, and examining factors for vaccine uptake such as demographics, parental knowledge, social support, information, and beliefs regarding vaccines. The exclusion criteria consisted of studies conducted in a hospital setting and among doctors with children, as this subpopulation’s vaccine perception and needs may differ from the general population. In addition, articles that did not show factors related to HPV vaccine uptake were excluded. Two reviewers (M.L. and H.P.) conducted title/abstract screening and full-text screening independently, and any disagreements were resolved by the third reviewer (E.S.).

### 2.2. Analysis

After identifying relevant articles, the following data were extracted for analysis: title and date of the study, study period, study design, sample size, and objective. Finally, the key research themes were identified in an iterative process.

## 3. Results

### 3.1. Search Results and Survey Characteristics

**Figure 1:**
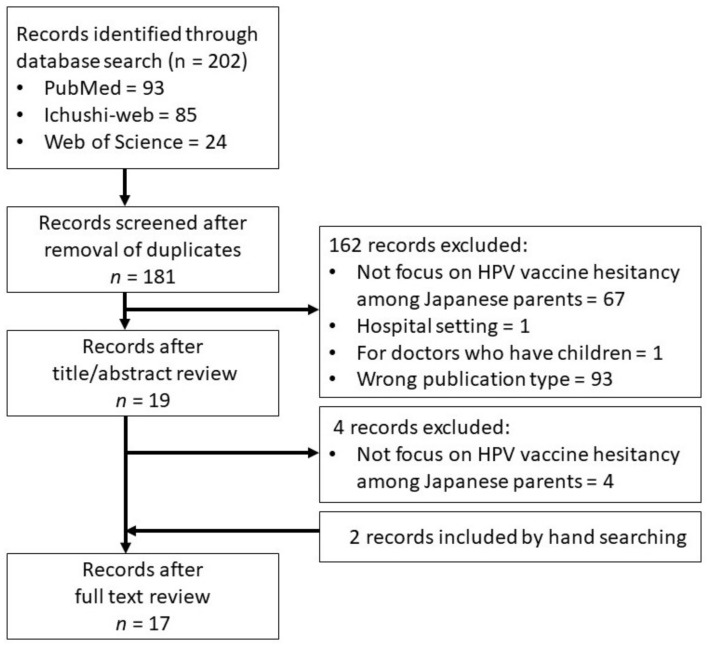
Flowchart of the selection process.

The study selection process is shown in Figure 1. Seventeen studies met the criteria; study characteristics and main factors of HPV vaccine uptake are summarized in Table 2. Two included studies were qualitative, and two articles were written in Japanese. Although the article by Shuto et al. [25] included mothers, female adolescents, and healthcare professionals, it was included as factors associated with vaccine uptake were reported separately. Some articles [26,27,28,29] were included even though they involved an intervention, as factors for vaccination were investigated separately.

Four key themes which affected HPV vaccine hesitancy and acceptance were identified: perceptions of risk and benefits, trust and recommendation, information and knowledge, and sociodemographic characteristics. The following subsections describe the details of these findings.

### 3.2. Perceptions of Risk and Benefits

Seven studies showed that safety concerns were a barrier to vaccine uptake [25,26,30,33,34,38,39]. For example, parents not willing to vaccinate perceived that the risk of side effects was higher than parents in the willing group [34]. In addition, some parents wanted their children to be inoculated only after many children in the same generation were vaccinated [39]. Parents had unarticulated anxiety caused by the short time since vaccine approval in Japan [33]. 

The effectiveness of vaccines was found to influence parental willingness for immunization [25,34,35]. Parents who wanted their children vaccinated believed vaccines were necessary to protect children’s health from HPV infection and cervical cancer [30]. Additionally, they estimated that the protection rate from cervical cancer was more than 60%, higher than parents who were not willing to vaccinate [34]. 

The perceived severity of the disease being vaccinated against was a promoting factor [30,34]. For example, if parents perceived a threat to children’s health from HPV infection, they accepted vaccination [30]. Additionally, perceived susceptibility to the disease was a significant factor in four studies [25,30,32,34,41]. For instance, in Egawa-Takata’s study [34], vaccination was desired when parents believed there was a 50% or greater chance that their children would get cervical cancer in their 20s. However, one study did not show any significance for the perception of susceptibility [38].

### 3.3. Trust and Recommendation

Vaccine information provided by the government was a positive factor [25,26,27,30,34,39,41]. On the other hand, the withdrawal of proactive recommendations for the HPV vaccine by the MHLW had a clear negative association with vaccination decision making [37,38]. While some parents were more likely to get the vaccination if the government resumed the recommendation [27,39], others did not get the HPV vaccine even after restarting the proactive recommendation [34].

Several studies demonstrated that healthcare provider (HCPs) recommendations promoted vaccine uptake [25,26,27,30,32,38,39]. Information from HCPs given through the school system was also important [33]. Additionally, vaccine acceptance was higher when parents had social support from family or relatives, not only HCPs [33,38]. Intention to vaccinate was also promoted when parents thought friends had a positive attitude toward getting vaccinated [30,38]. For example, Yagi et al. [41] noted that the vaccination status of the daughters’ best friends was a more decisive factor than the governmental recommendation, even though it was not evidence-based.

### 3.4. Information and Knowledge

Strong parental knowledge of HPV infection [27,30,35,38], HPV vaccine [33,38], and cervical cancer [26,27,33,38] were associated with their willingness to vaccinate children. Parents required sufficient information about HPV [31,32] and the vaccination [25,31]. Parents who understood the importance of vaccination at a young age were more likely to get vaccinated [35]. Information about side effects and preventing side effects of vaccination gave parents a sense of security [27,40]. In addition, a lack of information on the vaccine’s effectiveness was associated with parental vaccine hesitancy [35].

### 3.5. Sociodemographic Characteristics

Fathers were likelier to have a positive intention for HPV vaccination than mothers [27,28]. According to occupation, parents who are not healthcare workers tend to hesitate to get vaccinated [25,38]. Other positive factors associated with vaccination were high school completion [38] and mothers with cervical cancer screening [34,36]. However, Hanley et al. [30] did not report any significance for these factors.

Good mother–daughter relationships and discussions were positively associated with vaccine uptake [33]. Although mothers and daughters hesitated to discuss HPV vaccination with fathers, HPV vaccine acceptance was not different by marital status, including single fatherhood [32]. Both fathers and mothers expressed difficulty discussing sexual and reproductive health issues relating to vaccination, which contributed to the choice not to vaccinate [33,35]. On the other hand, when parents had the opportunity to talk with each other about HPV vaccination, they were more willing to get their children inoculated [26,33]. 

## 4. Discussion

This paper reviewed the factors associated with HPV vaccination acceptance and hesitancy among Japanese parents for their children. We identified four factors: perceptions of risk and benefits, trust and recommendation, information and knowledge, and sociodemographic characteristics associated with vaccine uptake. In addition, this review identified aspects that merit further investigation.

Among the four factors identified, perceptions of risk and benefits were most commonly mentioned in the literature, with low safety concerns and awareness of the effectiveness of the HPV vaccine associated with parental vaccine acceptance in line with findings of previous reviews [42]. In general, vaccine-hesitant parents were more worried about the risks of the vaccine rather than the VPD [43]. On the other hand, rigorous education about HPV vaccine-preventable diseases for parents might lead to improved vaccination rates [44]. Moreover, this review shows that the perceived severity and susceptibility to the disease were also important factors for vaccine uptake. From these points, interventions focusing on HPV vaccine-preventable diseases and the risk of HPV infection may improve parental intention to inoculate their children. However, no studies showed significant results through intervention in the Japanese parental context.

Governmental recommendations influenced parents’ decision-making. In this regard, some parents were unwilling to get the vaccination, even after the MHLW resumed proactive recommendations for the HPV vaccination in November 2021 [34]. Although some studies suggest that compulsory vaccination may increase parental suspicion [43,45], there is precedence in Japan for the increase in vaccination rates after government recommendations. In 2014, the varicella vaccine gained routine vaccine status from voluntary vaccine status. As a result, the number of varicella-related hospitalizations among those <5 years began to decline in 2015, and by 2017, pediatric sentinel sites reported an 88.2% drop in varicella infections among 1–4-year-olds [46].

In addition to building trust around vaccines, including a vaccine in the routine immunization program has at least two other distinct advantages to counteract vaccine hesitancy. First, parents avoid out-of-pocket payments, a crucial driver of vaccine hesitancy [1], as vaccines in the routine immunization program are entirely subsidized. Secondly, including a vaccine under the routine immunization program allows parents to receive vaccine-related information on time to utilize public subsidies [35] and, therefore, may eliminate some of the challenges with HPV misinformation in Japan [41]. Okuhara et al. [47] reported the presence of more anti-HPV-vaccination online sites than pro sites when negative reports about the HPV vaccine spread in Japan. Ireland and Denmark, for instance, have successfully recovered the HPV vaccination rate through government-led remedial campaigns against the spread of false information [48,49]. Although there is no national commitment against vaccine misinformation in Japan, some organizations, such as the JSOG, are trying to address the problem. Therefore governmental recommendations must be combined with efforts to ensure parents trust the vaccination policy [50] and practice transparency in policymaking decisions [44].

In line with the recommendation of an earlier study on the HPV vaccine [17], we observed that some studies meeting our inclusion criteria reported that advice from healthcare professionals is a significant determinant of vaccine uptake. Similarly, one previous paper showed that parents believed the information and recommendations provided by HCPs, especially doctors, were one of the most reliable sources of information [51]. However, several researchers in Japan have highlighted the need for sensitization and training for HCPs, as some HCPs may hold anti-vaccine sentiments about HPV in Japan [8,17]. HCPs should be trained to provide accurate and timely information and actively communicate with parents [17,51]. Although this review focused on parental HPV vaccine hesitancy, it may be worthwhile for future studies to investigate HCPs’ HPV vaccine hesitancy as well.

HCPs can also play a significant role in addressing vaccine misinformation, albeit additional training may be necessary. Okuhara et al. [47] noted that anti-HPV-vaccination online messages were easier to read than the pro sites written by health professionals, the readability of which tends to be more difficult than the recommended fifth to sixth-grade level or lower [52]. In addition, minimal emphasis or training, if any, is provided for vaccine advocacy and public health message writing in Japanese medical schools [53]. Moreover, there is a paucity of research on patient educational materials in Japan. In the one study we could identify, the Japan Pediatric Society created and distributed a Vaccine Information Statement (VIS) for four recommended vaccines, and while this led to an overall improvement in vaccine-related knowledge scores, vaccination rates, and adherence rates for the first dose for the four vaccines remained similar to those who had not received the VIS [54]. 

In any case, Japanese parents hoped to acquire more information [25,31,32,35], and policymakers and HCPs should consider how to provide information in an easy-to-understand manner more carefully. For example, considering that the physiology of cervical cancer did not affect parental willingness [40], while the morbidity of cervical cancer did [26], parents might want the information to understand the risks intuitively. Several studies included in this review underscored that Japanese parents with a high-level understanding and sufficient knowledge about cervical cancer, HPV infection, and the vaccine did not hesitate to get their children vaccinated [26,27,30,35,38]. 

Improving parental health and vaccine literacy would be particularly important given that Japanese health literacy is relatively lower, for instance, than Europeans [55], and there is a strong media influence. As noted by Ueda et al. [56], after adverse events with HPV vaccination were reported in 2013, Japanese newspapers adopted a negative stand giving little consideration to the safety and effectiveness of data published by the WHO, the Japan Pediatric Society, and the JSOG. To avoid being misled by such superficial information, improving parental health literacy and actively providing information from the government and HCPs could be important. Better health literacy will also be essential to address the widespread antipathy among Japanese parents toward discussing sexual contexts for the HPV vaccine uptake [32,33,35].

### Limitations

There are some limitations to this review. First, we only used three search engines that indexed the peer-reviewed literature, and the grey literature was not searched. Moreover, due to the 17 included studies’ methodology, key determinants for uptake may have been overlooked. For example, participants in studies included in this review were mainly mothers. Therefore, paternal factors such as education level and knowledge of vaccine uptake for children remain unclear. Finally, the studies included in this review focused on parents who have daughters; therefore, the findings may be potentially different if parents with a son were included. It is noteworthy that even though the HPV vaccine is recommended for boys globally, studies on HPV vaccine hesitancy in boys are sparse. In Japan, HPV vaccine coverage is appreciably low in boys [57].

## 5. Conclusions

In conclusion, while governmental and HCPs’ recommendations are important factors, efforts to improve parental trust in the vaccine are required. Future interventions to counteract HPV vaccine hesitancy should actively disseminate information on vaccine safety and effectiveness, along with information on the severity and susceptibility of HPV infection. Additionally, more consideration should be given to better ways of providing information from policymakers and improving the health literacy of parents. Finally, there are merits to including fathers and guardians of boys in future studies.

## Figures and Tables

**Table 1:** Search terms used for the literature search.

#1 vaccine	“vaccin” [Supplementary Concept] OR “vaccin” [All Fields] OR “vaccination” [MeSH Terms] OR “vaccination” [All Fields] OR “vaccinable” [All Fields] OR “vaccinal” [All Fields] OR “vaccinate” [All Fields] OR “vaccinated” [All Fields] OR “vaccinates” [All Fields] OR “vaccinating” [All Fields] OR “vaccinations” [All Fields] OR “vaccination’s” [All Fields] OR “vaccinator” [All Fields] OR “vaccinators” [All Fields] OR “vaccine’s” [All Fields] OR “vaccined” [All Fields] OR “vaccines” [MeSH Terms] OR “vaccines” [All Fields] OR “vaccine” [All Fields] OR “vaccins” [All Fields]
#2 parents	“parent’s” [All Fields] OR “parentally” [All Fields] OR “parentals” [All Fields] OR “parented” [All Fields] OR “parenting” [MeSH Terms] OR “parenting” [All Fields] OR “parents” [MeSH Terms] OR “parents” [All Fields] OR “parent” [All Fields] OR “parental” [All Fields]
#3 children	“child” [MeSH Terms] OR “child” [All Fields] OR “children” [All Fields] OR “child’s” [All Fields] OR “children’s” [All Fields] OR “childrens” [All Fields] OR “childs” [All Fields]
#4 Japan	“japan” [MeSH Terms] OR “japan” [All Fields] OR “japan’s” [All Fields] OR “japans” [All Fields]
#5 HPV	“HPV” [All Fields]
#6 human papilloma virus	“papillomaviridae” [MeSH Terms] OR “papillomaviridae” [All Fields] OR (“human” [All Fields] AND “papilloma” [All Fields] AND “virus” [All Fields]) OR “human papilloma virus” [All Fields]
#1 AND (#2 OR #3) AND #4 AND (#5 OR #6)	

**Table 2:** Summary of included articles on human papillomavirus (HPV) vaccination.

Author, Year	Study Characteristics				Objective	Key Findings (Theme ^#^)
	Period	Design	Methodology	Sample		
Hanley et al., 2012 [30]	2010	Cross-sectional	Paper questionnaire	862 participants; all mothers who had a daughters	To determine acceptance of and preferences for the HPV vaccine, examine attitudes toward HPV and its vaccine, and identify sociodemographic and attitudinal predictors.	High perception of susceptibility and severity of HPV promoted vaccination, while concern about side effects was a negative factor (***RB***) Recommendations from a doctor and local health board were important (***TR***). Parents who had heard of the HPV vaccine were more likely to opt for vaccination (***IK***).
Shida et al., 2015 [31]	2012	Qualitative	Content analysis of free text writing in a questionnaire	272 participants; parents/guardians who had a daughter	To explore what kind of information parents seek for their decision-making.	Parents needed information about HPV, cervical cancer, and the protective effect, side effects, long-term safety after administration, and limitations of the vaccine (***IK***).
Hanley et al., 2014 [32]	2010	Cross-sectional	Paper questionnaire	27 participants; all fathers who had a daughters	To investigate differences in vaccine acceptance in public funding programs and marital status.	No differences by marital status in perceptions of vaccine efficacy and safety. Single fathers thought their daughters were at risk for HPV and cervical cancer more than those who were married (***SDC***).
Nishigaki et al., 2014 [33]	2011–2012	Qualitative	Interview	20 participants; mother-daughter dyads	To examine vaccine uptake factors for mothers who have adolescent daughters.	Concerns about side effects were a negative factor (***RB***). Positive information from daughters increased willingness to vaccinate (***TR***). High health literacy and sufficient information about the HPV vaccine and cervical cancer of mothers and daughters promoted vaccination. Additionally, explanations from the school were important (***IK***). Goodmother–daughter relationships were positively associated with vaccine uptake (***SDC***).
Egawa-Takata et al., 2015 [34]	Not provided	Cross-sectional	Internet survey	1000 participants; all mothers who had a daughter	To investigate the frequency of continuing/discontinuing HPV vaccination, how mothers influenced the decision, and mothers’ thoughts about a future HPV vaccination.	Parental perceptions of risk vs. benefits determined if daughters were vaccinated or not (***RB***). Mothers’ knowledge of the effectiveness of the HPV vaccine was important in promoting vaccination (***IK***).
Nakajima et al., 2015 [35]	2010–2011	Cross-sectional	Paper questionnaire	224 participants; all mothers who had a daughter	To identify awareness and decision-making factors.	Awareness of the danger of HPV infection was related to vaccine uptake (***RB***). Children of parents who understood the effectiveness at a young age were more likely to get vaccinated (***IK***).
Egawa-Takata et al., 2016 [36]	2015	Cross-sectional	Internet survey	618 participants; all mothers who had a daughter	To examine the social factors for advising daughters about the HPV vaccine and cervical cancer screening.	Mothers who got recent screening had a more favorable position for their daughter’s HPV vaccination than those without recent screening (***SDC***).
Yagi et al., 2018 [37]	2014, 2015, and 2016	Cross-sectional	Internet survey in three phases	200 (1st survey), 2060 (2nd survey), and 2000 (3rd survey) participants; all mothers who had a daughter	To examine the time-dependent relationship between the mothers’ willingness to vaccinate their daughters and government recommendations.	The suspension of the governmental HPV vaccine recommendation triggered mothers’ vaccine hesitancy (***TR***).
Shuto et al., 2021 [25]	2019	Cross-sectional	Internet survey	1646 participants; all mothers who had a daughter	To understand HPV vaccine confidence and willingness among mothers with at least one daughter aged 12–16, female adolescents, and healthcare providers.	Awareness of the seriousness of cervical cancer and the effectiveness or safety of the HPV vaccine were important factors (***RB***). Sufficient vaccine-related information was required (***IK***). Mothers’ HPV vaccine confidence was lower than HCPs (***SDC***).
Kobayashi et al., 2020 [38]	2017	Cross-sectional	Paper questionnaire	246 participants; parents/guardians who had a daughter	To analyze how government policy influenced parental HPV vaccine acceptance for their daughters and associated factors.	When parents had low perceived barriers against the HPV vaccine, they were more likely to get vaccination (***RB***). The government’s recommendation influenced parents’ decision-making. Support from family or relatives was also an important factor (***TR***). Good parental knowledge about cervical cancer and the HPV vaccine promoted vaccination (***IK***). Parents employed as healthcare workers or who completed high school education were more likely to get vaccinated (***SDC***).
Miyoshi et al., 2020 [26]	2017	Interventional	Internet survey	1648 participants; all fathers who had a daughter	To identify fathers’ role in a young girl’s vaccination decision-making and the effectiveness of an educational intervention to change their attitude towards the HPV vaccine.	Education with an information sheet did not improve the father’s willingness to get the vaccination for their daughters. Concerns about side effects caused hesitancy (***RB***). Recommendations from their doctors, schools, and local/national governments were important (***TR***). Better recognition of the morbidity of cervical cancer was important to improve vaccination (***IK***). Having chances to talk with wives about vaccination for daughters was important (***SDC***).
Egawa-Tanaka et al., 2020 [27]	2018	RCT	Via Internet	1499 participants; all mothers who had a daughter	To determine whether mothers’ willingness would change intervention by a letter recommending talking with their husbands about HPV vaccination and/or an educational leaflet.To identify the mothers’ decision-making process.	Father’s participation in decision-making did not influence the mother’s willingness to vaccinate. After intervention with leaflet(s), factors such as a resumption of the governmental recommendation and the opinions from doctors became significantly important (***TR***). After intervention with leaflet(s), mothers thought information and knowledge about the risk of cervical cancer and preventing the side effects of the HPV vaccine were more important (***IK***).
Suzuki et al., 2021 [28]	2018	RCT	Via Internet	1660 participants; parents who had a daughter or son	To assess the effectiveness of a web-based educational intervention for parental decision-making.	A brief web-based educational intervention enhanced especially father’s willingness to vaccinate. Fathers were more willing to consider vaccination for daughters and sons than mothers (***SDC***).
Ugumori et al., 2021 [39]	2020	Cross-sectional and follow-up	Questionnaire	59 participants; all mothers who had a daughter	To investigate the mothers’ attitudes before and after the doctor’s explanation about the information leaflet.	Some mothers hesitated to vaccinate for daughters because of persistent safety concerns (***RB***). Some mothers were more willing to get the vaccination if governmental recommendations resumed (***TR***). Doctors’ explanations increased parental willingness (***IK***).
Imanishi et al., 2022 [40]	2021	Cross-sectional	Paper questionnaire	161 participants; all mothers who had a daughter	To clarify the effectiveness of doctors’ explanation about vaccine safety by the leaflet.	Knowledge of possible adverse events and specific solutions to them improved parental willingness. However, the pathology of cervical cancer and the HPV vaccination process was unimportant (***IK***).
Suzuki et al., 2022 [29]	2020	RCT	Via Internet	2175 participants; parents who had a daughter	To investigate the effect of a cervical cancer survivor’s story on parents’ decision-making based on examining the rate of parents’ vaccine acceptance and vaccination rate at three months.	The intervention increased parental willingness after three months but did not increase vaccination rates. Watching a cervical cancer survivor’s film influenced mainly fathers’ positive intentions but was not associated with vaccination rates (***IK***).
Yagi et al., 2022 [41]	2021	Cross-sectional	Internet survey	1576 participants; all mothers who had a daughter	To examine the mothers’ willingness to vaccinate themselves and their daughters against HPV and their reasons.	When mothers worried their daughters might get cervical cancer, they were more willing to get the vaccination (***RB***). “The daughter’s best friends were vaccinated before her” was the stronger factor than the local government recommendation (***TR***). When mothers had a positive attitude towards general vaccination, it promoted the HPV vaccination intention.

^#^ Themes: RB = perceptions of risk and benefits; TR = trust and recommendation; IK = information and knowledge; SDC = sociodemographic characters.

## Data Availability

Not applicable.
